# Acknowledging the gap: a systematic review of micronutrient supplementation in infants under six months of age

**DOI:** 10.12688/wellcomeopenres.16282.2

**Published:** 2023-02-20

**Authors:** Isabella Stelle, Sruthi Venkatesan, Karen Edmond, Sophie E. Moore

**Affiliations:** 1 Department of Women and Children's Health, King's College Hospital, London, Westminster Bridge Road, London, SE1 7EH, UK; 2 Nutrition Unit, MRC Unit The Gambia at London School of Hygiene and Tropical Medicine, Banjul, The Gambia

**Keywords:** Iron, Iron Deficiency, Iron Deficiency Anaemia, Anaemia, Multiple Micronutrients, Micronutrients, Infants, Supplementation

## Abstract

**Background**: Micronutrient deficiencies remain common worldwide, but the consequences to growth and development in early infancy (under six months of age) are not fully understood. We present a systematic review of micronutrient interventions in term infants under six months of age, with a specific focus on iron supplementation.

**Methods**: We searched the Cochrane Central Register of Controlled Trials (CENTRAL), MEDLINE (Ovid) and Embase (Ovid) from January 1980 through December 2019. Interventions included iron or multiple micronutrients (MMNs).

**Results**: Of 11,109 records identified, 33 publications from 24 trials were included (19 iron and five MMN supplementation trials). All but one trial (evaluating only morbidity and mortality) evaluated the effect of supplementation on biochemical outcomes, ten reported on growth, 15 on morbidity and/or mortality and six on neuro-behavioural development. Low- and middle- income countries made up 88% (22/25) of the total trial locations. Meta-analysis was not possible due to extensive heterogeneity in both exposure and outcome measures.  However, these trials indicated that infants less than six months of age benefit biochemically from early supplementation with iron, but the effect of additional nutrients or MMNs, along with the impacts on growth, morbidity and/or mortality, and neuro-behavioural outcomes remain unclear.

**Conclusions**: Infants less than six months of age appear to benefit biochemically from micronutrient supplementation. However, well-powered randomised controlled trials are required to determine whether routine supplementation with iron or MMNs containing iron should commence before six months of life in exclusively breast-fed infants in low-resource settings.

## Introduction

Iron deficiency anaemia (IDA) is estimated to affect 1.2 billion people worldwide ^1^. Overt IDA is associated with increased risk of impaired immunity, serious morbidity, poor pregnancy outcomes and poor motor function and mental development in children ^2^. It remains the leading cause of years lived with disability in low- and middle-income countries (LMICs) and is responsible for more than 120,000 maternal deaths per year ^3^. The prevalence of anaemia is five times higher in LMICs than high-income countries (HICs) and globally about 43% of children between the ages of six to 59 months are reported to be anaemic ^3, 4^. As such, IDA is the largest nutritional deficiency disorder worldwide and one of the five leading contributors to the global disease burden ^3^.

To date there are global and national level policies and guidelines around iron supplementation in women of reproductive age and young children. For all women of reproductive age, in areas with an IDA prevalence over 40%, the World Health Organisation (WHO) recommends daily iron supplementation (30 – 60 mg of elemental iron) for three consecutive months per year ^5^. During pregnancy, regardless of IDA rates, the addition of folic acid is recommended through the use of daily oral iron folic acid (IFA) supplementation (30 - 60 mg of elemental iron and 0.4 mg of folic acid) to prevent maternal anaemia, puerperal sepsis, low birth weight (LBW), and preterm (PT) birth ^6^. If pregnant women find daily IFA unacceptable due to side effects, and when the anaemia prevalence is less than 20% among pregnant women, intermittent oral IFA supplementation (120 mg of elemental iron and 2.8 mg of folic acid) once weekly is recommended ^6^. For young children between the age of six and 23 months, in areas with an IDA prevalence over 40%, the WHO recommends daily iron supplementation (10 – 12.5 mg elemental iron) for three consecutive months per year ^7^. In infants under six months of age, exclusive breast feeding (EBF) is the only practice recommended to prevent anaemia ^8^. However, despite several widespread supplementation schemes, IDA prevalence has remained in the top five leading causes of death worldwide since 1990 ^3^.

This lack of change in IDA prevalence is significant, as micronutrient deficiencies during pregnancy and lactation have implications for infant nutritional status ^9^. When maternal diets are habitually low in micronutrients, infants are at risk of poor status in early life by consequence of a reduced endowment during foetal life coupled with – for some micronutrients – low levels in human milk ^10– 12^. Such undernutrition during the first 1000 days from conception until two years of age can cause irreversible damage to growth and cognitive development ^13^. This is especially relevant in LMICs, where one third of children fail to reach their developmental milestones by school age ^14^.

Sufficient iron is essential in pregnancy as iron stores for the first few months of life are passed from mother to infant in utero ^15^. In healthy, full-term infants the iron endowment accumulates in the foetal liver, stored as ferritin ^16^. The iron received in utero is intended to support growth and development in the first six months of life, acting as a buffer for the needs of new tissue formation. This is especially pertinent in countries where breast feeding is an infant’s main nutrition source, as human milk is very low in iron ^17^. Iron supplementation for deficient mothers during gestation has only been shown to improve maternal anaemia and increases neonatal birthweight, while reducing the risk of PT birth ^18^. However, the long-term efficacy of antenatal iron to benefit infant iron status has not been proven ^18^.

A recent analysis of 317 rural Gambian infants, from an area where EBF rates are high, found infants were born with a reasonable endowment of iron despite being born to mothers with high levels of deficiency ^19, 20^. However, following birth there was a rapid deterioration of both haemoglobin (Hb) and ferritin ^19^. By five months of age, about 95% of infants had serum iron levels below the clinical reference range and this continued beyond the first year of life ^19^. These data suggest that exclusively breast-fed infants, born in low-resource settings, are at risk of iron deficiency (ID) in the first six months. This leaves these infants vulnerable, as this age group is not addressed by policy recommendations beyond EBF ^8^.

Adding to the burden of IDA is the confusion around the best iron therapy regarding safety and effectiveness ^21^. Oral iron may not be the best route and the timing of interventions, if not in pregnancy and early infancy, may be too late to impact on early brain development ^22^.

It is known that undernutrition significantly contributes to chronic inflammation by weakening immune function and increasing susceptibility to recurrent and persistent infections (e.g. diarrhoea and upper respiratory tract infections) ^23^. Likewise, environmental enteric dysfunction (EED), a syndrome caused by frequent bacteria transmission through the faecal-oral route due to poor sanitation, is characterised by chronic inflammation and morphological changes in the small intestine (i.e. villous blunting), further contributing to malabsoprtion ^24^. However, unabsorbed dietary iron through fortification or supplementation has been shown to negatively impact the gut microbiota ^25^. It has also been shown that four hours after consumption of an iron tablet by adults (2 mg/kg ferrous sulphate), human blood greatly supports enhanced rates of replication of pathogenic bacteria ^26^. This leads to reduced resistance to infection, higher prevalence of diarrhoea and increased faecal calprotectin, a marker of gut inflammation ^27^. However, there is a lack of sufficient data on side effects from iron supplementation in infants and children to negate their benefits in low-resource settings ^28^.

Intermittent oral iron supplementation in children under 12 years of age has been systematically reviewed, but none of the included trials were conducted in infants under six months of age ^28^. The authors concluded that intermittent iron supplementation “improve[d] haemoglobin concentrations and reduce[d] the risk of having anaemia or iron deficiency in children younger than 12 years of age when compared with a placebo or no intervention” ^28^. It was noted, however, that information on mortality, morbidity, developmental outcomes and side effects are still lacking ^29^.

Further, maternal acquired immunity to malaria begins to decline in infants around three months of age and, in malaria endemic settings, iron supplementation in children has been shown to cause harm ^30, 31^. However, a 2016 Cochrane review by Neuberger and colleagues assessed oral iron supplements for children in malaria-endemic areas and found that if malaria prevention and management services are offered, there was no increased risk to clinical malaria from iron supplementation ^29^.

The WHO now may recommend the use of multiple micronutrients (MMNs) containing iron over IFA alone in regions where the benefits outweigh the disadvantages ^6^. There is strong evidence that maternal supplementation with MMNs during pregnancy has positive impacts on several birth outcomes, but a positive impact on infant iron status has not been shown ^32^. Likewise, there was insufficient data of MMN supplementation during lactation to report any impact on infant iron status ^33^. In pre-school age children, point of use fortification with micronutrient powders (MNPs) has been shown to reduce anaemia and ID ^34^. Recently published data also concluded that, in infants under two years of age, MNPs are better than no intervention and placebo, and may be as effective as daily iron supplementation ^35^. However, none of the included trials were in infants under six months of age and further research is needed to determine developmental outcomes ^35^.

Given the lack of policy addressing additional iron needs in infants under six months of age, in order to understand both the short and longer-term impacts of supplementation with iron or MMNs in this age group, we present here a systematic review of MMN supplementation in infants under six months of age, with a specific focus on iron.

## Objectives

### Primary objective

To examine the effects of iron, iron containing MMNs and/or MMNs during the first six months of life on infant outcomes (biochemical, growth, morbidity and/or mortality, and neuro-behavioural development).

## Methods

The Preferred Reporting Items for Systematic Review and Meta-analysis (PRISMA) guidelines were used ^36^. The PRISMA Checklist is presented in *Extended data* file 1 ^37^. The review protocol was registered on PROSPERO (registration number CRD42020165641, 11 ^th^ February 2020).

### Search methods

On January 21 ^st^, 2020, the Cochrane Central Register of Controlled Trials (CENTRAL), MEDLINE (Ovid) and Embase (Ovid) were searched without geographical limitations for resources from 1980 through 2019.

Clinical trials registries for ongoing or recently completed trials were also searched ( clinicaltrials.gov; controlled-trials.com; and who.int/ictrp).

MeSH search terms included: Iron; Iron, Dietary; Anaemia, Iron‐Deficiency; Folic Acid; Dietary Supplements; Trace Elements; Ferric Compounds; Ferrous Compounds; Micronutrients; Drug Administration Schedule; Dose‐Response Relationship, Drug; Time Factors; Infancy. The full search strategy for this systematic review can be found in *Extended data* file 2 ^37^.

### Exclusion & inclusion criteria

Trials were included if they supplemented infants with iron, MMNs (> two micronutrients) containing iron or MMNs not containing iron - noting that iron could be a control arm; were in infants under six months of age; or were randomised controlled trials (RCTs).

Trials were excluded if supplementation was initiated in all infants after six months of age; fortified or complementary formulas and/or foods were outlined in the protocol for use before six months of age; or they were trial protocols, quasi experimental trials, observational and exploratory trials, case trials, economic evaluations, programme reports, clinical charting, conference proceedings, letters to the editor, opinion papers or editorials.

Trials conducted in infants over six months of age or in other languages other than English were later excluded. Trials of LBW or PT infants were later excluded due to extensive reviewing elsewhere ^38^.

### Selection of trials

Search results were uploaded to EndNote X9, where duplicates were removed. Titles and abstracts of publications retrieved using the search strategies were screened to identify trials that potentially met the inclusion criteria. Full texts of these publications were then reviewed.

In addition, previous reviews (including cross references) were searched, as well as checking reference lists of identified trials for further relevant trials.

Trial selection was carried out by two reviewers independently (IS and SV) and then cross-checked. Any disagreements were discussed and resolved. To assess the entire scope of research conducted, no country was excluded.

### Data extraction, management & assessment of bias

Data was extracted into Microsoft Excel to aid extraction of relevant information from each included trial. A list of *a priori* variables to categorise and extract the data were used. Data extracted are included in *Extended data* file 5 ^37^.

The Cochrane Risk of Bias 2 (RoB 2) Tool ^39^, along with Review Manager 5 (RevMan 2011) ^40^, were used to assess bias and create associated tables and figures. No trials were excluded from the narrative synthesis based on their quality assessment.

## Results

**Figure 1.**
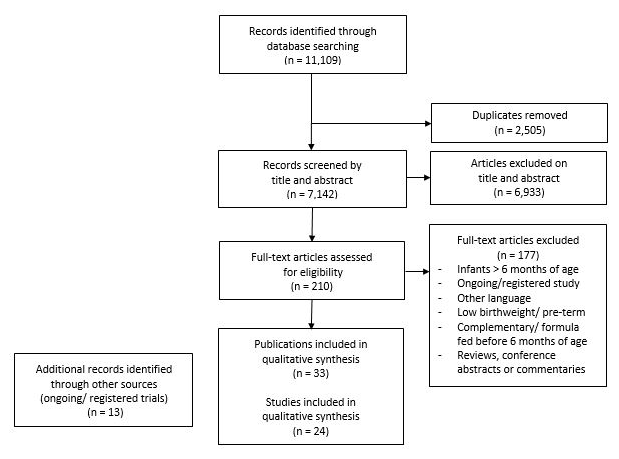
Selection of trials using the PRISMA flow chart.

The search identified 11,109 publications for possible inclusion, 2,505 of which were duplicate references ( Figure 1). 210 full text articles were reviewed for eligibility. Of these, 108 articles were excluded because they assessed LBW or PT infants, 28 because the full texts were not in English, 19 because further reading identified the age groups as over six months, 12 because they either used formula or complementary foods before six months of age as part of trial protocol, eight because they were reviews, one because it was a commentary and one because it was a conference abstract with full data published elsewhere. 33 publications, consisting of 24 trials, were retained for qualitative analysis by outcome. A further 13 references (trial protocols and registered and on-going trials) were identified through additional resources, but do not make up the 24 remaining trials for analysis ( *Extended data* file 3 ^37^).

For risk of bias analysis, of the 33 publications, six publications showed a low risk, 25 unclear risk and nine high risk of bias ( *Extended data* file 4 ^37^). Those showing unclear risk of bias were found to have small sample sizes (N < 100), unclear specifications on randomisation and/or mother’s supplementing the infants themselves. The trials with a high risk of bias had a lack of specifications on randomisation, a high dropout/non-compliance rate and/or lack of data reporting ^41^.

### Trial characteristics and outcomes

Nineteen iron and five MMN supplementation trials published between 1997 and 2019 were included in this review, with findings published across 33 separate papers. Sample sizes varied widely across the trials, from 70 to 94,359 infants and children, with data from 133,391 infants and children for analysis in this review.

**Figure 2.**
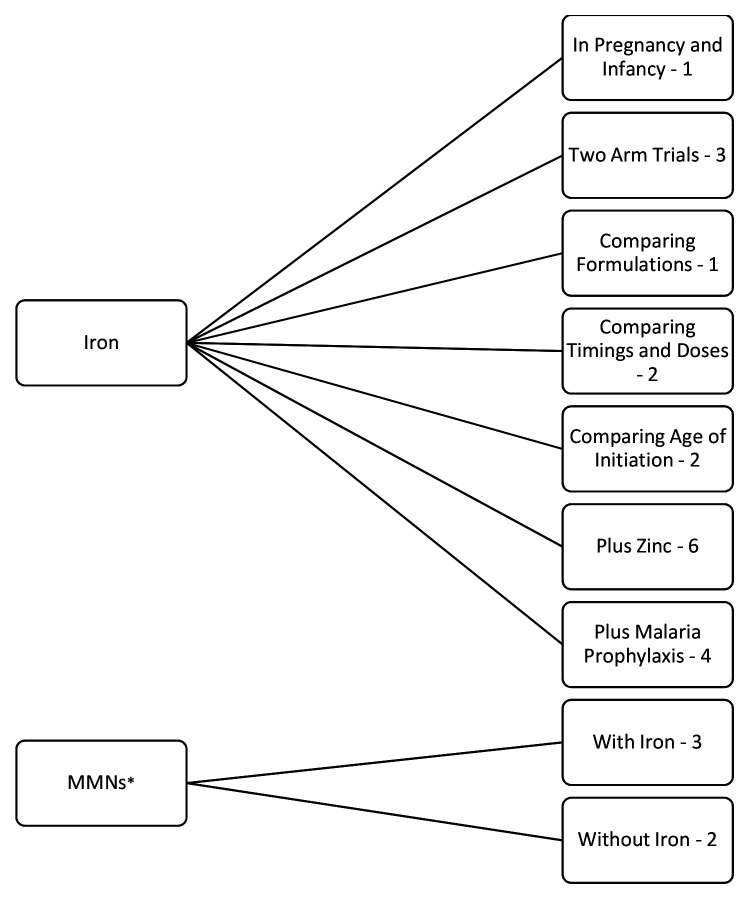
Flowchart of results grouped by trial design with the number of applicable trials (24 included in this review). *MMNs = multiple micronutrients.

Below, the results are reported by outcome: 1. biochemical, 2. growth, 3. morbidity and/or mortality, and 4. neuro-behavioural development. They are then further sub-divided by iron supplementation trials and MMN trials ( Figure 2). The iron supplementation trials are further subdivided into trials of supplementation in pregnancy and infancy, generic two-arm iron versus placebo trials, comparing formulations, comparing timings and doses, comparing age of initiation, with added zinc or with added malaria prophylaxis. The MMN supplementation trials are further subdivided into those with and those without iron.

Three of the five MMN trials gave MMNs also containing iron. All but one trial (evaluating only morbidity and mortality) evaluated the effect of supplementation on biochemical outcomes, ten additionally reported on growth, 15 on morbidity and/or mortality and six on neuro-behavioural development. Further trial characteristics and results, grouped by outcome, are provided as *Extended data* file 5 ^37^.

### Outcome 1: Biochemical

All but one trial ^42^ reported on biochemical outcomes for infants. Eighteen of these assessed supplementations with iron and five with MMNs. One of the iron trials additionally randomised mothers to iron or placebo in pregnancy ^43^. Of the trials assessing iron supplementation, four also included malaria prophylaxis in their interventions. Three of the five MMN trials included iron.

***Iron supplementation – in pregnancy and infancy.*** An RCT in rural China (N = 1,276) supplemented mother-infant pairs in pregnancy from 16 weeks gestation with daily ferrous sulphate or placebo, and infants from six weeks until nine months of age with daily iron proteinsuccinylate or placebo ^43^. Iron supplementation in infancy, but not pregnancy, reduced ID risk in infants at nine months of age, but more than 60% of infants still had ID at this age ^43^. However, effects of iron supplementation in pregnancy were observed when higher amounts of iron were distributed in infancy ^43^.

***Iron supplementation – two arm trials.*** A trial conducted in Northeast India randomised 200 infants (1/2 born to anaemic mothers) at 36 hours after birth until six months of age to daily ferrous ascorbate drops or standard of care ^44^. Infants born to anaemic mothers had significantly higher rates of ID at birth ^44^. Further, significantly higher Hb and serum ferritin (SF) in the supplemented versus placebo group were seen at six months of age, with comparable beneficial effects of supplementation in infants born to anaemic and non-anaemic mothers ^44^.

Another trial in rural India randomising 100 infants from four to six months of age for eight weeks to daily ferric ammonium citrate or placebo found significantly higher Hb and SF in the iron versus placebo group after eight weeks of supplementation ^41^. Likewise, the adjusted rise in Hb was higher in initially anaemic infants at the second follow-up ^41^.

A smaller (N = 77) two-arm trial in a low-income Canadian population found that infants randomised at one until six months of age to daily ferrous sulphate syrup or placebo had significantly higher Hb and mean corpuscular value (MCV) at six months of age versus the placebo group, but the significance was no longer seen at 12 months of age, six months following cessation of the intervention ^45^. There was a decline in SF over time; however, the decline was less in the iron supplemented group ^45^. At six months of age, infants in the iron group had significantly lower rates of IDA and ID ^45^.

***Iron supplementation – comparing formulations.*** An RCT testing ferrous sulphate drops versus ferric polymaltose drops in 112 Turkish infants from four until nine months of age found a significant increase in iron markers at nine months of age for both groups, but with the ferrous sulphate group being significantly higher than the ferric polymaltose group ^46^. ID and IDA rates were lower in infants receiving ferrous sulphate versus ferric polymaltose ^46^.

***Iron supplementation – comparing timings and doses.*** Another RCT in Turkey tested ferrous sulphate drops daily or weekly versus a standard of care (control) group in 70 infants at four until seven months of age ^47^. Infants supplemented weekly had the lowest rates of ID and IDA ^47^. Infants whose mothers had ID or IDA during the trial period were more likely to develop ID or IDA ^47^. Likewise, SF levels decreased between four and six months of age in the control and daily iron group, while the weekly groups showed no such decrease ^47^. However, in all groups, the mean levels of SF were significantly increased during the weaning period ^47^. 

A third RCT in Turkey tested ferrous sulphate drops at different time intervals and quantities by giving 113 five month old infants iron at either 1 mg/kg/d; 2 mg/kg/d; or 2 mg/kg/48hours versus placebo until nine months of age ^48^. No ID was observed in all three iron groups at nine months of age and there were significant increases compared to baseline values for Hb, MCV and SF for all three iron groups ^48^. Further, MCV, SF and Hb were significantly higher in all three iron groups versus placebo, but MCV was higher in the 2 mg/kg/d group versus the other iron groups, and SF was significantly higher at nine versus five months of age in this group also ^48^.

***Iron supplementation – comparing age of initiation.*** A trial in the Republic of Benin supplemented 612 infants twice daily with ferrous fumarate powder at four (intervention group) or six to 18 months of age (control group), both for two months ^49^. After supplementation, there was a significant increase in mean Hb and mean corpuscular Hb, but no significant change in values of MCV in the intervention and control groups, respectively ^49^. A decreased anaemia prevalence from 42.6 to 33.8 % in the intervention and from 62 to 30.2 % in the control groups were also seen ^49^. In infants who were anaemic at the start of the trial, the Hb increase was higher than that of the whole population ^49^. 

One trial, conducted across two contrasting settings (Sweden (N = 101) and Honduras (N = 131)) compared three trial arms in infants from four until nine months of age, supplemented daily with either ferrous sulphate from four to nine months of age (early initiation group); placebo from four to six months of age and then ferrous sulphate from six to nine months of age (later initiation group); or only placebo from four to nine months of age ^50^. A statistically significant increase in Hb and SF was observed in the early initiation group in both countries versus both other groups at six months of age ^50^. A statistically significant increase was also seen in SF in both countries’ intervention groups versus placebo at nine months of age ^50^. However, only in infants from Honduras was there a statistically significant increase in Hb in supplemented groups versus placebo, along with a statistically significant lower IDA prevalence in the interventions groups, both at nine months of age ^50^. In Sweden, iron supplements caused no reduction in the already low prevalence of IDA at nine months of age ^50^.

***Iron supplementation – plus zinc.*** A trial supplemented 478 Indonesian infants at four until ten months of age with either iron, zinc, iron + zinc, or placebo syrups ^51^. Iron alone was more effective than iron + zinc in increasing Hb and SF and in reducing the prevalence of anaemia ^51^. Further, IDA prevalence was significantly lower in the iron and iron + zinc groups versus the zinc only and placebo groups ^51^.

A further trial supplemented 609 Thai infants at four to six months of age for six months with either ferrous sulphate, zinc sulphate, iron + zinc; or placebo syrups ^52^. Infants in the two groups not receiving iron had a decrease in Hb concentration between baseline and six months, while the overall effect of iron supplementation on end point Hb concentrations was an increase of 10.8 g/L ^52^. However, the effect of zinc supplementation with or without iron was a decrease in Hb concentrations ^52^. Likewise, after six months of supplementation, the two groups receiving iron supplementation (iron or iron + zinc) had significantly higher SF than those receiving only zinc or placebo ^52^. It was also observed that anaemia prevalence was significantly lower in infants receiving only iron than in infants receiving iron + zinc, only zinc or placebo ^52^. ID and IDA prevalence were lower in infants receiving iron or iron + zinc versus zinc only and placebo ^52^.

A third trial supplemented 915 Vietnamese infants at four until seven months of age, daily, with either iron, zinc, iron + zinc (doses as previous) or placebo syrups ^53^. Hb and SF were higher in both iron and iron + zinc groups compared to zinc and placebo groups ^53^.

A very large RCT tested daily IFA, IFA + zinc, or placebos supplements in 26,250 Nepalese infants at one to 35 until 36 months of age ^54^. The IFA containing groups of the trial were stopped early on the recommendation of the data and safety monitoring board ^54^. However, in a subsample, 12 months after the start of supplementation, Hb was highest in the IFA group and median SF was significantly higher in the IFA group than both other groups and significantly higher in the IFA + zinc containing group versus placebo ^54^. The prevalence of IDA was lowest in the IFA only group ^54^.

Finally, another very large RCT conducted in a low-middle socioeconomic neighbourhood of India randomising 94,359 infants at one to 23 months of age for 12 months, daily, to IFA or IFA + zinc found that % SF < 20 μg/L was marginally lower in the IFA group versus the IFA + zinc group ^55^.

***Iron supplementation – plus malaria prophylaxis.*** An RCT in Tanzania supplemented 832 infants from eight weeks until six months of age with iron and from eight weeks until 12 months of age with malaria prophylaxis. At the trial end point, combined iron supplementation and malaria prophylaxis was found to have a protective effect on severe anaemia when compared to infants that did not receive iron ^56^. A second publication from the above trial also found that iron supplemented infants had a significantly lower prevalence of ID at five, eight and 12 months of age, but ID did not differ between those who did and those who did not receive malaria prophylaxis at any time point ^56, 57^.

A second RCT in Tanzania supplemented 291 infants from 12 – 16 weeks of age for six months with either daily ferric ammonium citrate mixture; amodiaquine; daily iron; or double placebo ^58^. Enrolment took place during the time of year that infants were most vulnerable to malaria ^58^. Infants receiving malaria prophylaxis and malaria prophylaxis + iron were protected against anaemia, but those receiving only iron were only partly protected against anaemia ^58^.

A further trial in Tanzania compared 14 days of daily ferrous sulphate tablets + one dose of sulfadoxine-pyrimethamine (SP) on day one versus three months of daily ferrous sulphate + three SP doses at the start of each month in 311 infants from two months up to five years of age ^59^. Two weeks after completing treatment, the prevalence of PCV < 33% was higher in the 14-day versus three-month intervention group, with mean PCV significantly higher in the three-month versus 14-day treatment groups ^59^. However, there was no difference in the prevalence of PCV < 25% two weeks after supplementation and the benefits of the extended therapy were only apparent six months after recruitment ^59^.

A four-arm RCT randomised 546 Western Kenyan infants with mild anaemia, at two to 36 months of age, for 12 weeks, to either intermittent preventive treatment (IPT) with SP at four and eight weeks + ferrous sulphate drops; placebo IPT SP + iron; IPT SP + placebo iron; or double placebo ^60^. Mean Hb at 12 weeks was higher for all three supplemented groups versus placebo and daily iron in conjunction with malaria prophylaxis was most effective in treating mild anaemia, while the prevalence of severe anaemia was lowest for the IPT + iron group ^60^.

***MMNs supplementation – with iron.*** An RCT conducted in Indonesia randomised 387 infants from four months of age for six months to either ferrous sulphate, zinc sulphate, iron + zinc, beta-carotene, zinc + beta-carotene, or placebo syrups ^61^. Hb was higher in infants receiving iron only versus the zinc + iron group but significantly higher compared to those receiving only zinc, only beta-carotene, zinc + beta-carotene or placebo ^61^. Likewise, SF was highest in the group receiving iron and significantly higher in the iron + zinc versus placebo groups ^61^.

Another RCT supplemented Indonesian infants (N = 800) from three to six months of age for six months, daily, with either zinc sulphate, zinc + ferrous sulphate, zinc + iron + vitamin A, or placebo syrups ^62^. Only the MMN group was found to have an increase in Hb, whereas the zinc only and placebo groups had a significant decrease in Hb. Hb in the MMN and zinc + iron groups were significantly higher than the zinc only and placebo groups after six months of supplementation ^62^. Likewise, anaemia prevalence significantly increased in the zinc only and placebo groups, with the MMN arm being the only group to see a decrease after six months of supplementation ^62^.

One further RCT supplemented 75 American infants at one until 5.5 months of age, daily, with MMNs with or without iron ^63^. Infants were followed until 18 months of age. Infants receiving MMN with iron had significantly improved SF levels at four and 5.5 months of age compared to the non-iron group. However, SF decreased continuously throughout the trial in both groups and Hb concentration showed no difference at any age, although it was higher for the group receiving iron than those not during the supplementation period, but not afterwards ^63^. At 5.5 months of age, but not at other ages, plasma soluble transferrin receptor (sTfR) was significantly lower for the iron versus the non-iron group and MCV was significantly higher in the iron than the non-iron group at 7.5 and nine months of age ^63^.

***MMNs supplementation – without iron.*** 2,387 Tanzanian infants, born to HIV positive mothers, were part of a large RCT in which they were supplemented from six weeks of age for 24 months, daily, with a multivitamin or placebo ^64, 65^. Hb concentrations were significantly higher in the treatment versus placebo group at 12, 18, and 24 months ^65^. Compared to those in the placebo group, infants in the treatment group had a lower risk of anaemia ^65^. The treatment was associated with a reduced risk of severe anaemia among infants born to women without anaemia, but not among those born to women with anaemia ^65^.

One trial analysed results from 2,006 Tanzanians who were part of a RCT supplementing infants daily from six weeks of age for 18 months, with either zinc sulphate; multivitamins; zinc + multivitamins; or placebo ^66, 67^. Infants given multivitamins had higher mean Hb than those given placebo or zinc alone at 18 months ^67^. Multivitamins were also associated with lower odds of ID and a reduction in risk of severe anaemia through 18 months, whereas zinc was associated with greater odds of ID at six months of age and had no positive effect on severe anaemia through 18 months ^67^.

All trial designs have now been described and will not be repeated in the remaining three outcome sections below; trials will be described by country with the relevant references provided.

### Outcome 2: Growth

Ten trials assessed growth, seven of which supplemented with iron and three with MMNs. One of the iron trials also randomised mothers to iron or placebo in pregnancy ^43^. Two of the three MMN trials included iron.

***Iron supplementation – in pregnancy and infancy.*** The RCT in China supplementing mother-infant pairs across pregnancy and infancy found no statistically significant effects of supplementation on anthropometry at nine months of age and no differences were observed when comparing infants who were iron sufficient versus those who were deficient at birth ^43^. Between groups, weight gain was significantly lower in infants whose mothers received placebo in pregnancy and there were no group differences in length gain at nine months of age ^43^.

***Iron supplementation – two arm trials.*** The trial in rural India randomising infants from four to six months of age to iron or placebo found no significant difference in anthropometry after eight weeks of supplementation ^41^.

The trial in a low-income Canadian population with infants at one until six months of age, followed until 12 months of age, found no effect of iron therapy on anthropometry when iron and placebo were compared at any clinic visit ^45^.

***Iron supplementation – comparing formulations.*** An RCT in Turkey testing ferrous sulphate drops versus ferric polymaltose drops in infants from four until nine months of age, found similar increases for both groups for weight, length and head circumference at nine months of age ^46^.

***Iron supplementation – plus zinc.*** The trial in Indonesia supplementing infants at four until ten months of age with either iron, zinc, iron + zinc syrup, or placebo syrups found that during the trial z-scores decreased significantly in all groups, with no differences among the groups at the end of supplementation ^51^. 

The trial in Northeast Thailand supplementing infants at four to six months of age, daily, with either iron, zinc, iron + zinc, or placebo syrups found that after six months of supplementation, group length did not differentiate, but that when controlled by gender and birth weight, iron supplementation improved weight-for-length z-score (WLZ) whereas zinc did not ^52^.

A similar trial design, but conducted in Vietnam and recruiting infants from four to seven months of age found that weight gain was higher in the zinc versus iron + zinc, placebo and iron only groups at nine months of age ^53^. No significant effects of the different interventions on length at nine months of age were seen ^53^.

***MMNs supplementation – with iron.*** The RCT with infants from Indonesia who were supplemented from three to six months of age daily with either zinc, zinc + iron, zinc + iron + vitamin-A, or placebo syrups found no beneficial effect on the prevalence of stunting, wasting and underweight across all four groups after six months of supplementation. Additionally, stunting prevalence doubled between end of supplementation and six months later for all groups ^62^. However, in initially stunted infants their mean height-for-age z-scores (HAZ) decreased during the six months of supplementation and this inter-group difference became significantly larger in the zinc + iron and MMN groups but not the placebo and zinc only groups, after four months of supplementation ^62^.

The RCT supplementing infants in The United States at one month of age with daily MMNs with (intervention) or without (control) iron found that iron in addition to the MMNs had no significant effect on growth across the groups at 5.5 months of age ^63^.

***MMNs supplementation – without iron.*** The MMN trial in Tanzania supplementing infants at six weeks of age for 18 months daily with either zinc; multivitamins; zinc + multivitamins or placebo found that there were no significant differences in any of the growth biomarkers of infants who received zinc compared with those who did not, or of infants who received multivitamins compared to those who did not at any of the assessed time points (six and 12 months of age) ^68^. A further publication from the same trial highlighted that infants in all groups experienced growth faltering and that supplementation did not have a significant effect on mean change in HAZ or on rates of stunting, wasting, or underweight ^69^. Changes in weight-for-age z-score (WAZ) and WHZs were significantly different across the four groups, where the mean decline in the WAZ from baseline to the end of follow-up in the zinc + multivitamin group was significantly less than in the placebo group and the decline in the WHZ was significantly greater in the zinc-only group than in the placebo group ^69^.

### Outcome 3: Morbidity and/or mortality

Fifteen RCTs assessed infant and childhood morbidity and/or mortality as an outcome. Thirteen of these trials supplemented with iron and two with MMNs. One of the iron trials also randomised mothers to iron or placebo in pregnancy ^43^. Of the trials assessing iron supplementation, four also included malaria prophylaxis in their randomisation. None of the MMN trials included iron.

***Iron supplementation – in pregnancy and infancy.*** The large four-arm RCT supplementing mother-infant pairs in pregnancy and infancy in China found no significant group differences in hospitalization or illnesses by nine months of age ^43^.

***Iron supplementation – two arm trials.*** Indian infants randomised from four to six months of age for eight weeks to daily iron or placebo drops had no statistical difference in reported interval morbidity in the two groups, but black colouring of the stools was significantly higher in the iron-supplemented versus placebo group ^41^.

A second trial, from Northeast India, randomising infants (100 born to anaemic and 100 born to non-anaemic mothers) at 36 hours after birth until six months of age to daily iron drops or standard of care found no differences in the parental report of morbidities between groups ^44^.

***Iron supplementation – comparing formulations.*** When testing ferrous sulphate drops versus ferric polymaltose drops in Turkish infants from four until nine months of age, the prevalence of reported adverse effects was 34.8%, with no statistical difference between either group and with the most common side effects being gastrointestinal complaints such as diarrhoea, constipation, and vomiting ^46^.

***Iron supplementation – comparing timings and doses.*** The RCT testing daily or weekly iron drops compared to no treatment (control) in infants from Turkey at four until seven months of age found that side effects of iron supplementation occurred in 44.4% of the infants, with no significant difference between groups, but higher percentages in the weekly group ^47^.

***Iron supplementation – plus zinc.*** The trial conducted in Vietnam supplementing infants daily at four until nine months of age for six months with either iron, zinc, iron + zinc, or placebo syrup found no significant effects of supplementations on morbidity ^53^.

The very large RCT randomising infants from India at one to 23 months of age for 12 months, daily, to IFA or IFA + zinc found the overall death rates did not differ significantly between the two groups when adjusted for cluster randomisation and the addition of zinc to IFA did not affect adjusted hospitalizations rates overall or specific rate ratios for diarrhoea and pneumonia ^55^.

In a similar trial design, in Nepal, with the addition of placebo, in infants at one to 35 until 36 months of age, Tielsch and colleagues found that although the IFA containing arms had to be stopped early, the all-cause mortality between treatment groups did not differ ^54^. Likewise, there were no significant differences in the reported morbidities between groups ^54^.

The one RCT that did not have biochemical results for infants under six months of age supplemented infants from 1–36 months with IFA, IFA + zinc, zinc alone, or placebo. Results on adverse events (85/2135), morbidity (17/295), and hospital admissions (68/1840) were reported for infants under six months of age, but no conclusions could be drawn from this data due to enrolment numbers not being divided by age group ^42^. However, it is worth noting the iron and folic acid-containing groups of the trial were stopped early on Aug 19, 2003, on the recommendation of the data and safety monitoring board ^42^.

***Iron supplementation – plus malaria prophylaxis.*** The RCT conducted in 832 Tanzanian infants supplemented at eight weeks until six months of age with iron and until 12 months of age with malaria prophylaxis found that during the main trial period, iron supplementation had no effect on the frequency of malaria and groups that received malaria prophylaxis had lower frequencies of malaria ^56^. However, during the follow up period, after the intervention, from 12 to 24 months of age, infants who received malaria prophylaxis had higher rates of malaria than those not receiving malaria prophylaxis during their first year of life ^56^.

Another RCT in Tanzania supplementing infants vulnerable to malaria from 12–16 weeks of age for six months with either daily iron; amodiaquine; iron + amodiaquine; or double placebo found a protective effect of malaria prophylaxis in prevention of malaria fevers at the end of the intervention, and at the four month follow up, they did not show rebound morbidity ^58^.

The third trial in Tanzania comparing either 14 days of daily iron + one dose of SP versus three months of daily iron + three SP doses at the start of each month in infants from two months up to five years of age found no differences in morbidities between the two groups ^59^. The prevalence of *P. falciparum* parasitaemia was also similar in the two groups in both cross-sectional surveys ^59^.

The RCT in West Kenya randomising infants with mild anaemia at two to 36 months of age, for 12 weeks, to IPT SP at four and eight weeks + daily iron; placebo IPT SP + iron; IPT SP + placebo iron; or double placebo found no significant interaction between iron supplementation and malaria prophylaxis on the risk of malaria or non-malaria morbidity. Further, between four and 12 weeks after enrolment, IPT versus iron was associated with significant reductions in malaria parasitaemia and clinic visits and a nonsignificant reduction in clinical malaria ^60^.

***MMNs supplementation – without iron.*** The trial supplementing Tanzanian infants at six weeks of age for 18 months, daily, with either zinc; multivitamins; zinc + multivitamins; or placebo found a nonsignificant increase in all-cause mortality among infants who received zinc compared with those who did not ^64^. Further, multivitamins did not affect the occurrence of any of the morbidity symptoms ^66^. Likewise, there was no significant treatment effects of zinc or multivitamins on EED or systemic inflammation at six months of age ^66, 68^.

Another trial assessed Tanzanian infants, born to HIV positive mothers, who were supplemented at six weeks of age for 24 months with daily multivitamin or placebo ^64^. They found that morbidities were not significantly different between the groups, except for fever and vomiting which were significantly lower in the multivitamin group ^64^. Among 429 children whose mothers received antiretroviral therapy, multivitamin use had no effect on mortality but was associated with a significant reduction in morbidities ^64^.

A sub-analysis of infants from the previous RCT identified new cases of HIV infection during follow-up in 3.9% children in the placebo group and 4.7% in the multivitamin group with no effect of multivitamins on mother to child transmission ^64, 65^.

### Outcome 4: Neuro-behavioural development

Six RCTs assessed the impact of supplementation on neuro-behavioural developmental outcomes. Four of these supplemented with iron and two with MMNs. One of the iron trials also randomised mothers to iron or placebo in pregnancy ^70^. Neither of the MMN trials included iron. Only two of the trials looked at longer term developmental outcomes, one at six to eight and the other at nine years of age ^71, 72^. The remaining trials assessed outcomes between six and fifteen months of age.

***Iron supplementation – in pregnancy and infancy.*** The large RCT in China supplementing mother-infant pairs in pregnancy and infancy with daily iron or placebo followed 1,196 infants until nine months of age for developmental scores ^43, 70^. Iron supplementation in infancy but not pregnancy improved gross motor scores overall, but there were no group differences in overall neurologic integrity ^43, 70^.

***Iron supplementation – two arm trials.*** Northeast Indian infants (100 born to anaemic and 100 born to non-anaemic mothers) were randomised to daily iron or standard of care at 36 hours after birth until six months of age ^44^. Motor development was closer to age appropriate norms and their active and passive tone milestones were significantly higher in the iron supplemented group at six months of age ^44^.

The other trial, conducted in a low-income Canadian population, randomised infants at one until six months of age to daily iron or placebo ^45^. The iron supplementation group scored significantly higher in Bayley psychomotor developmental indices and tended toward improved visual acuity at 13 months of age, which became significant when the data were analysed excluding noncompliers ^45^.

***Iron supplementation – plus zinc.*** North-eastern Thai infants from a trial who were supplemented at four to six months of age for six months, daily, with either iron, zinc, iron + zinc, or placebo syrups were followed up with at nine years of age (N = 560) ^52, 72^. No significant differences in any of the neuro-developmental outcomes between the four groups were seen ^52, 72^. Further, school performance scores were not different between groups ^52, 72^.

***MMNs supplementation – without iron.*** Tanzanian infants (N = 365), from a trial, were followed up with at six and eight years of age to assess three developmental domains ^66, 71^. In the intervention trial, infants were randomised at six weeks of age for 18 months, to either daily, zinc; multivitamins; zinc + multivitamins; or placebo ^66, 71^. There was no effect of either zinc or multivitamin supplementation on any of the three developmental domains at six to eight years of age following the supplementation in infancy ^66, 71^.

A second MMN trial assessed 192 HIV negative Tanzanian infants at 15 months of age, who were born to HIV positive mothers and from a trial with supplementation at six weeks of age for 24 months with daily multivitamin or placebo ^64, 71^. No effect of MMN supplementation on any of the developmental domains at 15 months of age were seen, but there was a trend toward improved fine motor skills among infants in the MMN group ^73^.

### Countries represented in trials

**Figure 3.**
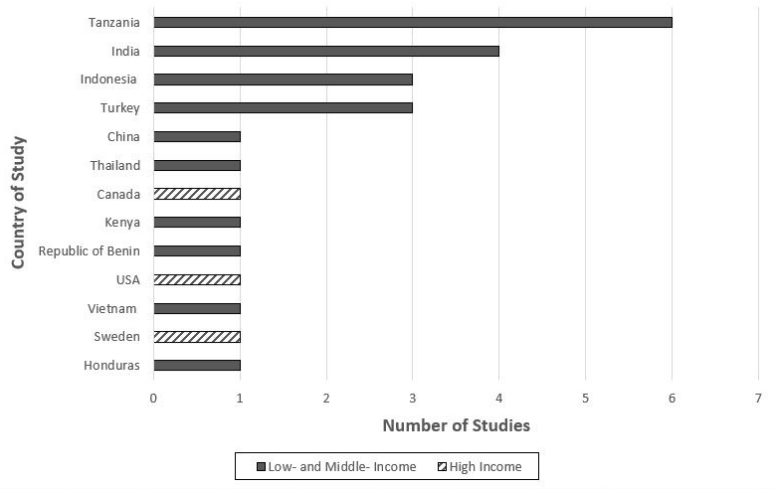
Distribution of countries included in this review. USA = United States of America.

Of the 24 trials, 13 countries were represented ( Figure 3); three HICs and ten LMICs. The majority of the trials took place in a single location, but one trial recruited half of its participants in a LMIC (Honduras) and the other half in a HIC (Sweden) ^50^. The two other HICs represented were Canada and the United States of America, but the Canadian trial was conducted in a low-income population ^63, 74^. LMICs made up 88% of the total trial locations (22/25). Five of the middle-income countries were middle-upper, constituting China, Thailand, and Turkey. Ten of the countries were low-middle, constituting Honduras, Kenya, India, Indonesia, and Vietnam. Seven were low-income, constituting Tanzania and Republic of Benin. 72% of the trials took place in Asia and sub-Saharan Africa (18/25), with 8/25 of the trials being in sub-Saharan Africa and 10/25 in Asia.

## Discussion

### Summary of main results

Infants living in settings with poor dietary diversity may be at risk of micronutrient deficiencies in early infancy (under six months of age); a period largely missed by policy guidelines, except for recommendations on EBF. This systematic review aimed to examine the effects of iron, iron containing MMNs and/or MMNs during the first six months of life on infant outcomes (biochemical, growth, morbidity and/or mortality, and neuro-behavioural development). We observed that infants less than six months of age benefit biochemically from early supplementation with iron, but the impacts of additional nutrients or MMNs, along with the impacts on growth, morbidity and/or mortality, and neuro-behavioural outcomes are less clear.

In infants less than six months of age, iron (alone or in combination with other nutrients) is effective in increasing Hb and SF concentrations and reducing the prevalence of anaemia. Positive impacts of supplementation were seen in infants from low resource settings in a HIC and in LMICs; however, in resource secure HICs the impact was not as significant. Responses may differ based on iron formulation and timing of doses, but data is scarce and therefore no clear conclusions can be drawn. Likewise, while biochemical status was improved with MMNs not containing iron, the effect is greater when MMNs contain iron. In comparison to iron and MMN supplementation, infants receiving zinc only are were more likely to develop anaemia.

The impact of supplementation on growth, morbidity and/or mortality and longer-term neuro-behavioural development was found to be mixed. For growth, no consistent beneficial effects of supplementation in early infancy were observed. In areas with a high prevalence of malaria, iron supplementation was effective in preventing severe anaemia without increasing susceptibility to malaria while treatment was on-going. While there were no significant increases in morbidity and/or mortality, gastrointestinal side effects were reported for infants receiving iron. One study did stop the iron and folic acid-containing groups on the recommendation of the data and safety monitoring board ^42^. However, while this study did report adverse events, mortality, and hospital admissions in infants under six months of age, enrolment data for this age group was missing, so no conclusions could be drawn ^42^. Some benefits were seen directly after supplementation with MMNs (without iron) and with iron only, but longer-term impacts were less clear after supplementation with MMNs (without iron) and iron and/or zinc. However, the evidence on neuro-behavioural outcomes is limited and, therefore, no firm conclusions can be drawn.

Finally, it was consistently observed that supplementation must continue past six months of age, because any beneficial impacts seen across all outcomes and supplementations disappear when supplementation ceases. A further detailed review of the results, by outcome, follows.

### Effects of supplementation on infant biochemical outcomes

Of the nine trials assessing biochemical outcomes and supplementing with iron only versus placebo (or none), all nine showed a beneficial effect for infant biochemical outcomes ^41, 43– 50^. From these trials, the following further themes emerged. First, supplements administered directly to the infants outperformed supplementation administered to mothers during pregnancy, although, this observation was limited to a single trial ^43^. Second, the impact of supplementation was greater among infants who were anaemic at the start of the trial ^49, 50^. Third, iron formulation may impact on efficacy, with ferrous sulphate performing better than ferric polymaltose, although again, this observation was limited to a single trial ^46^. Fourth, continued supplementation during the weaning period is beneficial ^45, 48^. Lastly, while increasing dose (e.g. 2 versus 1 mg/kg) improved outcomes ^48^, the impact of frequency of dosing was less clear; in one trial daily supplementation outperformed alternate day supplementation ^48^, but in another trial weekly supplementation was found to outperform daily supplementation ^48^. It must be noted that trials considering dose and timing had small sample sizes (N = 70 - 113) ^45– 48^.

A further finding was that supplementation with additional micronutrients did not enhance infant iron status. For example, trials that included zinc or beta carotene (either alone or in combination with iron) showed no added benefit over iron alone ^51– 55, 61, 62^. Likewise, MMNs without iron are not as beneficial as those with iron ^63^, but are more beneficial than a placebo ^64^ or zinc alone ^66^ on infant iron status. Another emerging theme was that infants born to mothers with ID or IDA were more likely to benefit from supplementation ^64^.

In comparison, all trials of iron and/or malaria prophylaxis found iron supplementation in combination with malaria prophylaxis to be more protective against anaemia than iron alone ^56– 58, 60^. In addition, long-term iron supplementation and malaria prophylaxes (three months) is more beneficial than short-term (14 days), although, this observation was limited to a single trial ^59^.

### Effects of supplementation on infant growth outcomes

The beneficial effects of supplementation in the first six months of life for infant growth were less evident. From four trials supplementing with iron only versus placebo (or none) and assessing growth outcomes (N = 1,565), no beneficial effects were observed for infant growth outcomes ^41, 43, 45, 46^. Further, supplementation with additional micronutrients had an equivocal impact on infant growth. In all trials with additional micronutrients, there was no benefit to infant growth of the additional micronutrients on growth markers ^51, 52, 62, 68, 69^, except for one trial reporting zinc supplementation as more beneficial for weight gain only ^53^. Additionally, in all trials assessing growth, all infants experienced growth faltering ^51– 53, 62, 68, 69^. Further themes emerged from the trials reporting growth outcomes. First, stunting prevalence increased more than two-fold between the end of MMN supplementation (including iron) and a six-month follow up, with initially stunted infants at even greater risk ^62^. Second, when controlled for gender and birth weight, iron only may improve WLZ over zinc only supplementation ^52^. Last, one trial found a lower decrease in WHZ and WAZ in multivitamins (without iron) versus placebo supplementation ^69^. However, all three of these findings were limited to single trials ^52, 62, 69^. Further, with only a single trial (N = 112) comparing iron formulations, no conclusions can be drawn ^46^.

### Effects of supplementation on infant morbidity and/or mortality outcomes

The beneficial effects of supplementation in the first six months of life on morbidity and/or mortality were equivocal in their findings. Of the five trials assessing these outcomes and supplementing with iron only versus placebo (or none), all showed no significant differences for infant outcomes ^41, 43, 44, 46, 47^. In view of the concerns regarding the potential negative consequences of iron supplementation ^23– 27^ it is relevant that no differences were observed with respect to iron formulation ^46^, but weekly supplementation did result in fewer side effects ^47^. However, these observations were limited to single trials, with small sample sizes (N = 112 and 70) ^46, 47^.

With respect to combining iron with other micronutrients, supplementation with additional zinc did not affect infant morbidity and/or mortality outcomes ^53– 55^. One trial had to stop the iron and folic-acid containing arms of the trial early, on recommendation of the data and safety monitoring board, however conclusions could not be drawn from this trial due to lack of missing recruitment data for infants under six months of age ^42^. However, except for EED and systemic inflammation for which there were no differences ^68^, MMNs (without iron) may be more beneficial than zinc alone or placebo supplementation on other morbidity and/or mortality outcomes such as hospitalizations or unscheduled outpatient visits ^65– 67^.

Trials of iron supplementation in combination with malaria prophylaxis found, firstly, that malaria prophylaxis does not negatively interact with iron supplementation ^60^. Secondly, long-term iron supplementation and malaria prophylaxes (three months) may not be more beneficial than short-term (14 days) for morbidity and/or mortality ^59^, but when supplementation stops, infants may be at higher risk of malaria than previously ^56^. However, both observations were limited to single trials.

### Effects of supplementation on neuro-behavioural outcomes

All three trials assessing neuro-behavioural outcomes and supplementing with iron only versus placebo showed significant differences for some of the short-term infant outcomes assessed ^44, 45, 70^, suggesting that iron, in the short-term, may improve neuro development in early infancy ^44, 46, 70^. Supplementation with MMNs (without iron) was suggested to improve fine motor skills in the short term (single trial, N = 192 infants assessed at 24 months of age) ^73^, but little evidence was found to support longer term effects at six to eight years of age ^71^.

### Explanatory factors

Given the multi-factorial nature of supplementation in infants and children, various factors may influence the heterogeneity in the results of some outcomes. For biochemical outcomes, it is apparent that iron supplementation in infants under six months of age has a beneficial effect on infant iron status ^41, 43– 50^. However, the impact on growth, morbidity and/or mortality, and neuro-behavioural development is less conclusive. For growth, initially stunted infants are at even greater risk of later stunting ^62^, so prior undernutrition may be an important predictor of response to supplementation. Further, in areas of malaria endemicity, iron supplementation in conjunction with malaria prophylaxis is more beneficial on infant iron status, indicating a potential interaction between infection control and iron supplementation ^56– 58, 60^. Lastly, while EBF up to six months of age was encouraged in all trials, it was an inclusion criteria for only two trials ^47, 48^. Likewise, only five of the trials reported on mean age of EBF ^43, 51, 52, 65, 67^. Therefore, while heterogeneity in rates of EBF could also be an explanatory factor, it precludes a sensitivity analysis by mode of feeding.

### Comparison to the literature

To our knowledge, this is the first systematic review to look at iron or MMN supplementation in infants under six months of age. A Cochrane protocol is registered to analyse daily iron supplementation for prevention or treatment of IDA in infants, children, and adolescents; however, it does not specify if this will include infants under six months of age ^75^. A handful of published systematic reviews, discussed below, of iron and/or MMN supplementation in infants and children have been conducted; however, none focused specifically on infants under six months of age ^29, 30, 35, 36, 76, 77^.

Intermittent iron supplementation (versus placebo or standard of care) improved biochemical markers of iron status in a study of children under 12 years of age ^28^, but the evidence to support the relative benefit of intermittent versus daily supplementation was less clear in our results ^41, 43– 50^. In the children, type of regimen, dose or composition had no biochemical impact ^28^, whereas one trial from our results found ferrous sulphate to be more effective than ferric polymaltose ^46^ and another found iron supplementation with 2 versus 1 mg/kg as more effective ^48^. Second, zinc supplementation in children aged six months to 12 years of age had no effect on Hb or iron status ^76^, which was consistent with our results ^51– 55^. However, in the children, the effects on growth showed a very small improvement in height and a small increased risk of death due to diarrhoea, lower respiratory tract infection or malaria ^76^, which is not consistent with our results ^51– 55, 76^. Third, in children under 18 years of age in areas at risk of malaria, iron and malaria prophylaxis were more protective against anaemia than iron alone and decreased the number of clinical admissions due to malaria ^29^, which was consistent with our results in young infants ^56– 58, 60^.

### Strengths and limitations

There were a limited number of trials available, with much heterogeneity in the exposure and outcome measures, to provide a comprehensive assessment of the impact of iron interventions early in life on growth, morbidity and/or mortality and neuro-behavioural development. Only six of the 24 trials were efficacy trials, in which infants were supplemented directly by health care workers rather than by caregivers. Further, eight of the 11 MMNs trials included did not include iron in their supplementation, but still reported on biochemical markers in relation to iron. Data for longer term neuro-behavioural development was limited, with only two trials assessing infants at either six to eight or nine years of age. Only one trial included supplementation to both mothers during pregnancy and then infants directly. Lastly, almost all trials took place in LMICs with only one low socio-economic group in a HIC.

The main strength of this systematic review is that is it the first comprehensive review of iron and/or MMN supplementation in infants under six months of age. In addition, trials were not excluded based on supplementation regimen or outcomes assessed, allowing the authors to understand a wide scope of the literature in the field.

We also acknowledge several limitations to our review. We purposefully only included RCTs, but a review of the full scope of literature, including observational trials, may have added insight. We excluded trials that used fortified or complementary formulas and/or supplementary foods before six months of age within the trial protocol to ensure a level of homogeneity across the data, as volumes and quantities consumed by subjects could vary widely. A further limitation is that most trials were not limited to young infancy, but included a wide age range from infants under six months of age and beyond. Also, only two studies specifically excluded non EBF infants. While heterogeneity in the data was a strength in terms of assessing the full scope of the literature, it made comparison of trials via a quantitative meta-analysis difficult.

## Conclusions

Growing evidence indicates that infants less than six months of age may be vulnerable to micronutrient deficiencies. This review shows that daily oral iron supplementation has beneficial effects on short-term biochemical outcomes such as iron status. Likewise, MMN formulations containing iron are more beneficial for iron status than those without. However, overall, short- and long-term evidence for this age group is limited. It is unclear whether iron supplementation in the first few months of life to exclusively breast-fed infants is beneficial across various outcomes and the longer-term growth and neuro-behavioural developmental outcomes are less clear. Evidence is lacking as to whether MMN formulations containing iron are more beneficial than iron alone and longer-term health and developmental impacts are also less clear. Well-powered randomised controlled trials are required to determine whether routine supplementation with iron or MMNs containing iron should commence before six months of life for exclusively breast-fed infants in low-resource settings.

## Data Availability

All data underlying the results are available as part of the article and no additional source data are required. Figshare: Acknowledging the gap: a systematic review of micronutrient supplementation in infants under 6 months of age_Extended Data File_Updated.docx. https://doi.org/10.6084/m9.figshare.22015367 ^37^. This project contains the following extended data: Extended datafile 1: PRISMA checklist. Extended datafile 2. Search terms. Extended datafile 3: Registered and ongoing trials. Extended datafile 4: Risk of bias analysis. Extended datafile 5: Table: Results by outcome. Figshare: PRIMSA checklist for ‘Acknowledging the gap: a systematic review of micronutrient supplementation in infants under six months’. https://doi.org/10.6084/m9.figshare.22015367 ^37^. Data are available under the terms of the Creative Commons Attribution 4.0 International license (CC-BY 4.0).
