# Supplementary material for: Acknowledging the gap: a systematic review of micronutrient supplementation in infants under six months of age
Source: Wellcome Open Res. 2023 Feb 20;5:238. Originally published 2020 Oct 12. [Version 2] doi: 10.12688/wellcomeopenres.16282.2 (PMC7713887; doi:10.12688/wellcomeopenres.16282.2)
Supplement: Peer review history

# Peer review: Reviewer response for version 2

**DOI:** 10.21956/wellcomeopenres.21080.r55058

**Published:** 2023-02-23

**Authors:** Peter Cooper

**Affiliations:** 1 Department of Paediatrics, Faculty of Clinical Sciences, University of the Witwatersrand, Johannesburg, South Africa

Are the rationale for, and objectives of, the Systematic Review clearly stated?

Yes

Is the statistical analysis and its interpretation appropriate?

Not applicable

Are sufficient details of the methods and analysis provided to allow replication by others?

Yes

Are the conclusions drawn adequately supported by the results presented in the review?

Yes

Reviewer Expertise:

Paediatrics, Neonatology, Nutrition

I confirm that I have read this submission and believe that I have an appropriate level of expertise to confirm that it is of an acceptable scientific standard.

# Peer review: Reviewer response for version 1

**DOI:** 10.21956/wellcomeopenres.17890.r41324

**Published:** 2020-12-02

**Authors:** Peter Cooper

**Affiliations:** 1 Department of Paediatrics, Faculty of Clinical Sciences, University of the Witwatersrand, Johannesburg, South Africa

Previous systematic reviews on micronutrient supplementation in infants and children have largely concentrated on those over six months of age. This would appear to be the first systematic review of micronutrient supplementation, with a specific focus on iron supplementation, in term infants under the age of six months and thus is an important contribution. 

 Standard guidelines for systematic reviews were followed and the methodology outlined is clearly described. Figure 1 illustrates that a total of 11,109 records were identified through database searching and 2,505 were removed as they were duplicates. It is not clear how 7,142 records then remained to be screened – *this needs to be clarified.*

 Their review identified only 32 publications from 23 studies and they have reported the findings in detail. However although their paper provides some answers, due the wide heterogeneity of these studies, many questions have been raised.

 Most of the studies were done in low and middle-income countries (LMIC) and not surprisingly showed a high incidence of iron deficiency in these infants, more particularly in those born to iron deficient mothers and thus clearly is a problem that requires to be addressed. Supplementation of iron fairly consistently in the studies reviewed resulted in improvement in iron status of the infants. There was little evidence from this systematic review that supplements with iron, zinc or other micronutrients resulted in improvement in growth of the infants or their morbidity and mortality. 

 There was some evidence that iron supplementation did contribute to improvements in measures of short-term neurodevelopment. Only two of the studies looked at longer term neurodevelopment up to six to eight and 11-14 years of age respectively. Neither of these studies showed any benefit but importantly iron supplementation was not included in their intervention arms so this remains an open question.

 This review also highlights the difficulties in doing studies of this nature. They were all done in the context that exclusive breast feeding was encouraged up to six months of age. However, the researchers of these studies were unable to control to what extent exclusive breast feeding took place and what additional foods may have been given. Commercially available infant foods and formulas often have supplements of iron and micronutrients, which may confound the results.

 The authors are to be congratulated for raising an important issue around which there has not been adequate research. Further large randomized studies are required and may need to be individualized to the country or countries in which they are done. For example, one of the studies in a malaria area that they analyzed showed that the addition of malaria prophylaxis to iron supplementation resulted in reduced prevalence of anaemia compared with iron supplementation alone. Studies done in other areas may need to take different local specific factors into account.

 This review will hopefully lead to the initiation of large randomized trials of supplementation of iron during the first six months of life. These studies need to take into account whether the infants are exclusively breast-fed or otherwise during the first six months of life and full dietary histories during this period need to be documented. Most importantly longer term follow up of haematologic status, growth, morbidity and mortality and neurodevelopment should be built into the study protocols.

Are the rationale for, and objectives of, the Systematic Review clearly stated?

Yes

Is the statistical analysis and its interpretation appropriate?

Not applicable

Are sufficient details of the methods and analysis provided to allow replication by others?

Yes

Are the conclusions drawn adequately supported by the results presented in the review?

Yes

Reviewer Expertise:

Paediatrics, Neonatology, Nutrition

I confirm that I have read this submission and believe that I have an appropriate level of expertise to confirm that it is of an acceptable scientific standard.

# Peer review: Reviewer response for version 1

**DOI:** 10.21956/wellcomeopenres.17890.r41347

**Published:** 2020-11-23

**Authors:** Filomena Gomes

**Affiliations:** 1 The New York Academy of Sciences, New York City, NY, USA

The manuscript describes an important, well conducted systematic review on iron and multiple micronutrient supplementation of infants under six months of age. PRISMA guidelines and a previously published protocol were followed. The authors did not perform a meta-analysis but presented the results in a balanced and informative way.

 A few minor comments:

- "Exclusion & inclusion criteria": the first sentence should have been more clear that the beginning of supplementation (not the whole supplementation or follow-up period) needs to happen in the first six months of life as an inclusion criteria. Instead of "Trials were included if (...) were in infants under six months of age", it could have been stated that "Trials were included if (...) supplementation was initiated in infants under six months of age"
- The method used to assess risk of bias is correct but the way the findings are described is imprecise. For example, instead of stating that 25 publications had an unclear risk of bias, it could have been stated that 25 publications had unclear risk of bias for the domain "blinding of participants and personnel" (which may be difficult to achieve in nutrition/diet interventions). In particular, authors are encouraged to report the overall judgment for the most critical domains, which usually include bias for sequence generation, allocation concealment and blinding of outcome assessors (particularly for subjective outcomes).
- Under "strengths and limitations", the second paragraph starts with "The main strength of this systematic review is that is it (...)" but should be "The main strength of this systematic review is that it is (...).

Are the rationale for, and objectives of, the Systematic Review clearly stated?

Yes

Is the statistical analysis and its interpretation appropriate?

Yes

Are sufficient details of the methods and analysis provided to allow replication by others?

Yes

Are the conclusions drawn adequately supported by the results presented in the review?

Yes

Reviewer Expertise:

Clinical nutrition, malnutrition, systematic reviews

I confirm that I have read this submission and believe that I have an appropriate level of expertise to confirm that it is of an acceptable scientific standard.
